# Stress hormones or general well-being are not altered in immune-deficient mice lacking either T- and B- lymphocytes or Interferon gamma signaling if kept under specific pathogen free housing conditions

**DOI:** 10.1371/journal.pone.0239231

**Published:** 2020-09-30

**Authors:** Sarah Jeuthe, Josephine Kemna, Christoph Philipp Kemna, Dario Zocholl, Robert Klopfleisch, Rupert Palme, Clemens Kirschbaum, Christa Thoene-Reineke, Thomas Kammertoens

**Affiliations:** 1 Animal Facility of the Max-Delbrück-Center for Molecular Medicine, Berlin, Germany; 2 Department of Gene Therapy and Molecular Immunology, Max-Delbrück-Center for Molecular Medicine, Berlin, Germany; 3 Institute of Immunology, Charité Campus Berlin Buch, Berlin, Germany; 4 Institut für Biometrie und Klinische Epidemiologie, Charité Campus Mitte, Berlin, Germany; 5 Department of Veterinary Medicine, Institute of Veterinary Pathology, Free University, Berlin, Germany; 6 Unit of Physiology, Pathophysiology and Experimental Endocrinology, Department of Biomedical Sciences, University of Veterinary Medicine, Vienna, Austria; 7 Department of Psychology, Technical University, Dresden, Germany; 8 Department of Veterinary Medicine, Institute for Animal Welfare, Animal Behavior and Laboratory Animal Science, Free University, Berlin, Germany; IGBMC/ICS, FRANCE

## Abstract

It is controversially discussed whether immune-deficient mice experience severity in the absence of infection. Because a comprehensive analysis of the well-being of immune-deficient mice under specific pathogen free conditions is missing, we used a multi-parametric test analyzing, corticosterone, weight, nest building and facial expression over a period of 9 month to determine the well-being of two immune-deficient mouse lines (recombination activating gene 2- and interferon gamma receptor-deficient mice). We do not find evidence for severity when comparing immune-deficient mice to their heterozygous immune-competent littermates. Our data challenge the assumption that immune-deficiency per se regardless of housing conditions causes severity. Based on our study we propose to use objective non-invasive parameters determined by laboratory animal science for decisions concerning severity of immune-deficient mice.

## Introduction

Mice with genetic alterations in immunologically relevant genes are frequently used in research. Whether such mice experience burden in the absence of infection, due to their complex phenotypes, is controversially discussed. Previously we found only minor differences in serum cytokine levels and the microbiota when comparing immune-deficient mice to heterozygous or wild type controls [1, 2]. However, other studies reported differences between immune-deficient and -competent mice based on physiological or behavioral alterations [3–7]. However, to our knowledge, a comprehensive analysis of the well-being of immune-deficient mice kept in standard, specific pathogen-free (SPF), housing conditions is lacking. In the current study we therefore investigated well-being of two immune-deficient mouse lines, one being deficient in recombination activating gene 2 (Rag^-/-^) resulting in lack of functional T cells and B cells and the other lacking expression of the interferon gamma receptor 1 gene (IFNγR^-/-^). Both genetic defects cause severe immune-deficiency in humans. We used a protocol that compared four different parameters (body weight, corticosterone, nest building and facial expression “Mouse Grimace Scale—MGS”) [8]. As refinement, in order to cause as little external stress as possible, we focused (with the exception of taking fur samples every three month) on non-invasive parameters that can be taken during weekly cage changes, without fixing the animal. Mice were observed over a period of nine months and immune-competent littermate mice served as controls (Main study protocol, S1 Table, S1 Fig). After nine months, mice were sacrificed and a pathological analysis of inner organs was performed.

## Materials and methods

The study was performed according to the guidelines of the German Animal Welfare Act and the Directive 2010/63/EU for the protection of animals used for scientific purposes and was approved by the Berlin State Authority (“Landesamt für Gesundheit und Soziales”, permit number: StN 0008/18). Data sets were acquired prospectively and analyzed for this study in a retrospective manner. We adhere to the Animal Research: Reporting of In Vivo Experiments (ARRIVE) guidelines. Research Institutes in which the study was performed (MDC and Charite) as well as first and last authors of the study have signed the Basel declaration. All staff involved in collecting the data in the main study protocol were blinded to the genotype of the mice.

### Mice

A total of 90 mice (genotypes Rag^-/-^; Rag^+/-^; IFNγR^-/-^; IFNγR^+/-^) at 7 to 40 weeks of age were subjected to the main study’s protocol involving body weight measurement, mouse grimace scale, corticosterone measurements of fur and nesting behavior (see S1 Table). Rag2-deficient mice (Rag2tm1.1Cgn/J) originally on C57BL/6J and crossed to an albino genetic background (C57BL/6Brd-Tyr^c-Brd^) and IFNγR1-deficient mice (B6.129S7-IFNγR1tm1Agt/J) on C57BL/6J genetic background were introduced by embryo transfer into our SPF breeding facility. In order to obtain immune-deficient (-/-) and heterozygous (+/-) immune-competent littermate controls for the study, male -/- were crossed to female +/- mice. Since there is, to our knowledge, no evidence of a gene dosage effect for either the Rag or the IFNγR1 transgene (i.e. the +/- animals are fully immune-competent) we decided to use the -/- x+/- breeding strategy based on 3R considerations (reduce). Because, if we had included the +/+ genotype, we would have had to use a +/- x+/- mating to get all 3 genotypes as littermates and we would have had to sacrifice surplus +/- mice. Samples collected from fur and feces at different time points did not always provide sufficient material for corticosterone analysis, thus not all animals could be analyzed at each time point in the respective groups. Homozygous and heterozygous mice of the main study were kept in separate cages during the experiment. No randomization into groups was performed, because we wanted to perform a longitudinal 9 months analysis, we decided to cohouse siblings of the same genotype in the same cage, as a refinement to minimize the chance that mice show aggressive behavior. The experimental groups were housed in similar cage stocking density (app. 3 mice per cage) during the main study. Specifically Rag^-/-^ males: 3 mice per cage (four cages, mice per cage 3, 4, 3, 2); Rag^+/-^ males: 2.8 mice per cage (four cages, mice per cage 3, 2, 3, 3); Rag^-/-^ females: 3.7 mice per cage (three cages, mice per cage 3, 4, 3); Rag^+/-^ females: 4 mice per cage (two cages, mice per cage 5, 3); IFNγR^-/-^ males: 2.5 mice per cage (four cages, mice per cage 3, 2, 3, 2); IFNγR^+/-^males: 2.8 mice per cage (five cages, mice per cage 3, 2, 3, 2, 4); IFNγR^-/-^ females: 3.3 mice per cage (three cages, mice per cage 4, 4, 2); IFNγR^+/-^ females: 3.5 mice per cage (four cages, mice per cage 4, 3, 5, 2).

In addition to the main study some samples (fur and/or feces) from other mouse strains (FVB/N, C57BL/6J, C3H/HeN) at single time points (no kinetic analysis) were used for corticosterone (metabolite) measurements. For these analyses FVB/N, C57BL/6J, C3H/HeN.Rag2^-/-^ and C3H/HeN.Rag^+/-^ were used, which were introduced by embryo transfer into our SPF breeding facility. FVB/N mice were included as internal controls in the pathology analysis, because C57BL/6 mice were reported to show strain specific pathology (e.g. in the kidney).

### Housing conditions

Animals used in this study were group-housed with two to five mice in individually ventilated cages (Tecniplast Germany GmbH, Green Line or Blue Line) and kept under identical housing conditions: 12h light/dark cycle (light cycle 6:30 a.m. to 6:30 p.m.), standard pelleted mouse diet (ssniff GmbH, Soest, Germany, article number v1124-300) ad libitum, free access to water, 22 ± 2°C room temperature and relative humidity of 55 ± 10%. The cages contained wooden bedding material (Tapvei Estonia OÜ, Estonia, Aspen bedding, article number 4HK 10 kg), nestlets (ssniff GmbH, Soest, Germany, article number H3279-10), a red plastic house (ZOONLAB GmbH, Castrop-Rauxel, Germany) and paper tunnels (ZOONLAB GmbH, Castrop-Rauxel, Germany; article number 3084030) as cage enrichment. Animals were handled by male and female caretakers and technicians.

### Mouse Grimace Scale (MGS)

MGS scores [9] were obtained weekly by four persons blinded to the experimental set up (typically one scientist, one technician, one caretaker and one veterinarian). Orbital tightening, nose bulge, cheek bulge, ear position, and whisker change were scored on a scale from 0 to 2 (0 = not present, 1 = moderately present, 2 = obviously present). Each of the facial action units were scored live, in order to reduce variability scoring was always performed between 9 and 12 a.m. A sum of the five action units was calculated per animal. In a first step this sum of all 5 action units was taken for each animal from each observer, the sums were than compared between the different groups, genotypes and sexes.

### Nest building behavior

The nest building behavior for cages with three to five mice was scored to detect changes in general behavior that might indicate changes in well-being as described previously [10]. The nests were scored using a modified protocol developed by Jirkof et.al. [10]. 8 nestlet patches (39 x 39 x 5 mm/patch, ssniff Spezialdiäten GmbH, Soest, Germany, article number H3279-10) each were placed in the cage. Three days later, the nests were photographed and nests building behavior assessed on a 5-point scale (1 = more than 90% of the nestlet intact; 2 = 50–90% intact; 3 = 50–90% shredded nestlet; 4 = more than 90% shredded but flat nest, i.e. less than 50% of its circumference being higher than the body height of a curled-up mouse; 5 = more than 90% shredded and high nest, i.e. more than 50% of its circumference being higher than the body height of a curled-up mouse). During scoring of the nests, the investigator was blinded to the genotype of the mice analyzed.

### Body weight

The body weight [g] was measured weekly for 25 weeks using a weighing scale.

### Fecal corticosterone metabolites

Fecal corticosterone metabolites–a non-invasive indicator of adrenocortical activity–were extracted (80% methanol) and measured in fecal samples with an established and validated enzyme immunoassay [for details see [11, 12]].

### Hair corticosterone and testosterone analysis

Hair corticosterone, as an indication for chronic stress was measured non-invasively for each animal after weaning, at the age of approximately 6 to 9 weeks and at 6 and at 9 months (the age distribution for taking fur in the individual groups is shown in S1 Table). Approximately 7.5 mg of hair from the abdomen (approximately 2 cm by 2 cm) was shaved with an electric shaver for small animals (Aesculap Isis GT 420, Suhl, Germany). The shaver was cleaned before and after use with paper tissues and disinfection solution. Hair corticosterone [pg/mg] was analyzed by liquid chromatography-mass spectrometry as described previously [13]. Testosterone analysis was also performed in parallel to corticosterone also using the same technique of liquid chromatography-mass spectrometry from the same samples.

### Histopathology

Mice were sacrificed by cervical dislocation and organs (spleen, heart, intestine, lung, liver and kidney) were fixed in 4% buffered formalin and then embedded in paraffin. Sections of each organ were stained with hematoxylin and eosin (HE) and analyzed by an experienced, board certified veterinary pathologist.

### Sample size

Using the TOST procedure in nquery advisor Version 8.5.2.0 a sample size calculation for a test for equivalence of two means was performed for the primary metric outcome corticosterone in fur. Assuming a standard deviation of 3 pg/mg, to achieve statistical power of at least 80% under type I error probability of 5%, a minimum sample size of 7 animals is required to detect equivalence up to a tolerance limit of 5 pg/mg. Because we wanted to perform an experiment with a 9 months observation we decided to aim for slightly larger groups aiming at a group size of at least 8 mice.

### Statistical analysis

**Fig 1:**
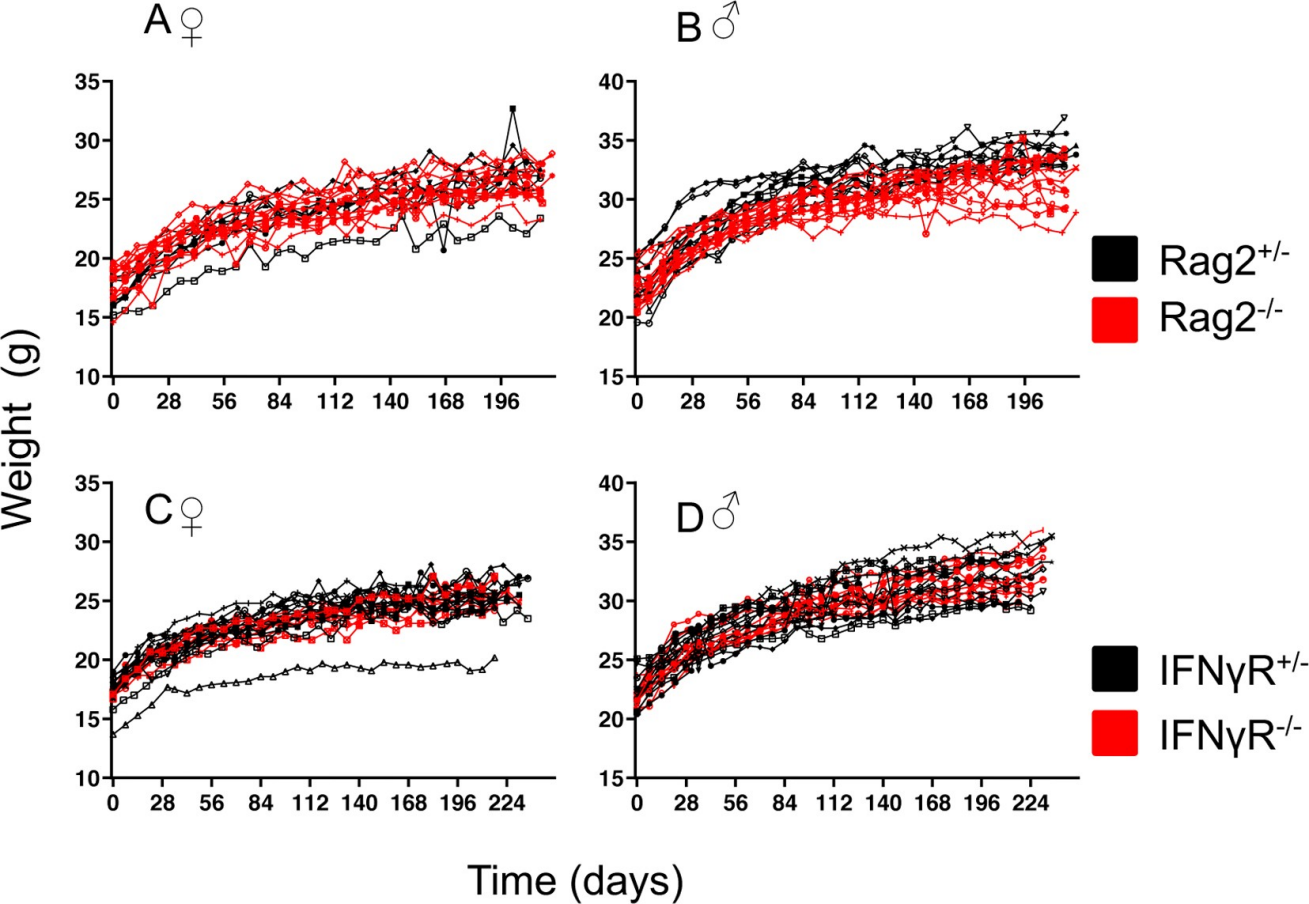
Weight development of immune-deficient and -competent mice. A, B Rag^+/-^ (black lines) and Rag^-/-^ (red lines), C, D IFNγR^+/-^ (black lines) and IFNγR ^-/-^ (red lines) mice were weighted weekly for nine months. Each line represents one mouse, A, C, female and B, D, male mice. Female Rag^+/-^ n = 8; female Rag^-/-^ n = 11; male Rag^-/-^ n = 12; male Rag^+/-^ n = 11; female IFNγR^-/-^ n = 10; female IFNγR^+/-^ n = 14; male IFNγR^-/-^ n = 10; male IFNγR^+/-^ n = 14. For statistical analysis see S2 Fig.

**Fig 2:**
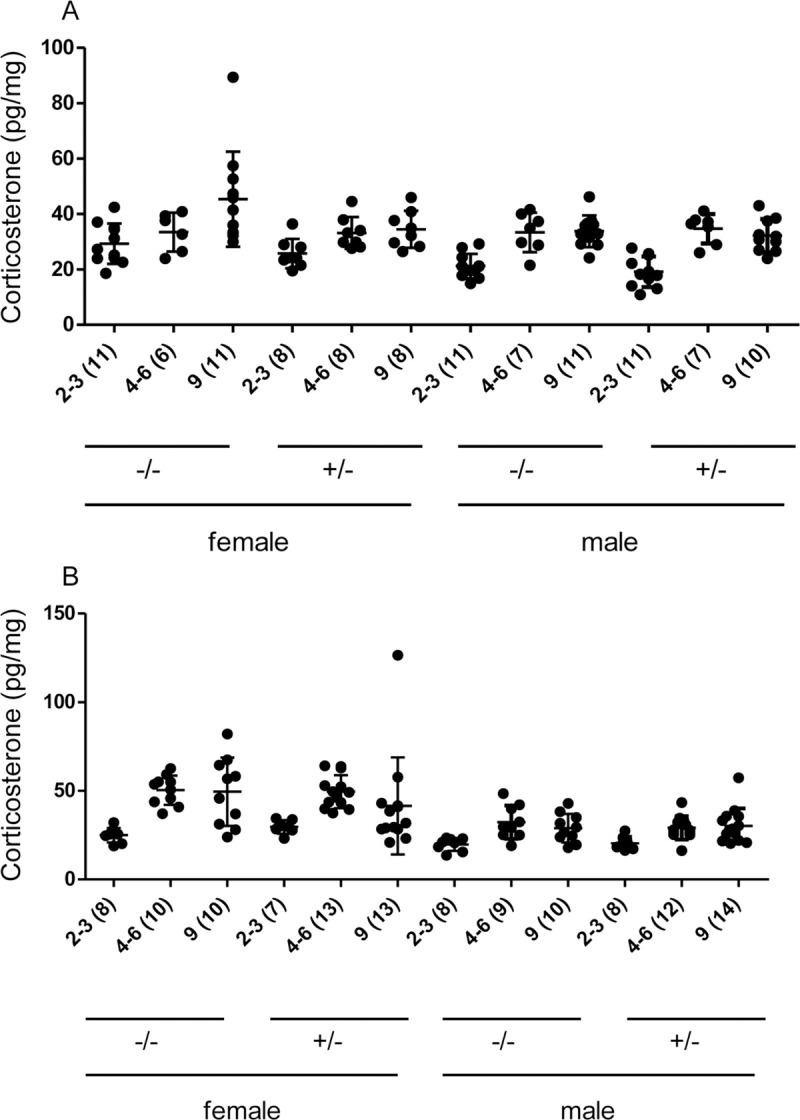
Corticosterone levels in immune-deficient and -competent mice. Fur samples of A, Rag^+/-^ and Rag^-/-^, B, IFNγR^+/-^ and IFNγR ^-/-^ mice were harvested after weaning (2–3 months), from young adults (4–6 months) or at 9 months of age and were analyzed for corticosterone by LC/MS. Each dot represents one mouse. Slight variations in sample numbers between the different time points were caused by the insufficient amount of fur for testing in some mice. Number of samples tested for each group is indicated in parentheses below the x-axis. For statistical analysis see S2 Fig.

All statistical analyses were performed in R version 3.6.2. To take repeated measurements into account, we implemented linear mixed effects models using the package nlme [14] and included individual mice as random effects. Graphics were produced in R using ggplot2 [15, 16] and in Graphpad Prism 5.0. The significance level was set to be 0.05. However, since some important conclusions of this study concern equivalence rather than difference, we report 95% confidence intervals in S2 Fig, and encourage the readers to focus on these instead of mere statistical significance. Particularly, the width of a confidence interval allows concluding whether an effect estimate was not significant because of large uncertainty or because the effect was actually so small that it can be considered as biologically irrelevant. Additionally we provide classical statistical significance testing with p-values for Figs 1 and 2 in S2 Fig.

## Results

Body weight analysis of immune-deficient (Rag^-/-^ or IFNγR^-/-^) and immune-competent littermate control mice (Rag^+/-^ or IFNγR^+/-^) revealed no evidence for significant differences (Fig 1A–1D, S2 Fig).

Similarly, analysis of long-term corticosterone levels measured in fur samples of the same mice after weaning (aged 2 to 3 months), in young adults (aged 4 to 6 months) and at nine months of age revealed neither statistically significant nor biologically relevant differences when comparing immune-deficient mice to their immune-competent littermate controls. Corroborating earlier studies that age and sex affect corticosterone levels in mice [17], we observed a moderate increase in corticosterone levels in older mice of all genotypes (Fig 2A and 2B, and S2 Fig) and found that female IFNγR mice of both genotypes (IFNγR^+/-^ and IFNγR^-/-^) exhibited slightly higher levels of corticosterone (Fig 2B and S2 Fig).

To further confirm that steroid hormones measured in fur samples can accurately reflect systemic levels, we also determined testosterone levels in both younger and older mice. As expected, a sex-specific difference of testosterone and an increase with age was observed (S3 Fig). To obtain information on behavior, nesting was analyzed and to monitor pain MGS was performed. Monthly analysis of nest building behavior revealed no evidence for differences between immune-deficient Rag^-/-^, IFNγR^-/-^ mice and Rag^+/-^, IFNγR^+/-^ controls (S4 Fig). MGS analysis revealed that very few mice showed slight symptoms (1.46% of all Rag^-/-^; 1.07% of all Rag^+/-^; 0.22% of all IFNγR^-/-^; and 0.16% of all IFNγR^+/-^ measurements). The slightly higher incidence of MGS signals in Rag mice may have been also affected by the fact that Rag mice on the albino background were used and fur color may affect scoring. Obvious symptoms were detected even more rarely with only one positive (out of 13920) test results for Rag^-/-^ and only 3 (out of 18220) test results for IFNγR^+/-^ mice (S2 Table). We consider that MGS analysis showed that the great majority of mice did not experience pain. Histopathologic analysis of liver, spleen, heart, kidney, lung and intestine at nine months of age found lesions mainly in kidney and less frequently in lung. Kidney lesions (chronic progressive nephropathy, CPN) were found more frequently in mice of C57BL/6 background (irrespective of immune-deficiency) than in FVB/N mice. In lung Pneumocyte Typ II-Hyperplasia (PT2H) had a similar distribution between the groups, Bronchus Associated Lymphoid tissue (BALT)—hyperplasia was more often observed in mice with IFNγR^+/-^ than with IFNγR^-/-^ genotype and acidophilic macrophage pneumonia (AMP) was observed in 6 out of 17 Rag^-/-^ but not in Rag^+/-^ mice. Together, pathological analysis revealed some age-related changes in the lungs and kidneys of mice but with the exception of 6 cases of AMP no difference between immune-deficient Rag^-/-^, IFNγR^-/-^ and immune-competent controls (Rag^+/-^, IFNγR^+/-^) was detected (S3 Table).

At nine months of age, 4 out of 11 female Rag^-/-^ mice showed a slight increase in corticosterone levels compared to heterozygous controls (Fig 2A). Therefore, we performed two further sets of corticosterone analyses in addition to the main study (S4 Table). In one set, fur samples of Rag^-/-^ and Rag^+/+^ mice were compared (non-control littermates from the breeding colony, at nine months of age) and no evidence for differences was found, also levels were comparable to wildtype mice on another inbred strain (FVB/N) (S5 Fig). In the second set, we analyzed both fur and feces of Rag^-/-^ and littermate Rag^+/-^ mice on a C3H genetic background. C3H mice were reported to produce higher levels of corticosterone when exposed to environmental stress factors [18]. When comparing Rag^-/-^ and Rag^+/-^ mice on the C3H genetic background, we found comparable levels of corticosterone (metabolites) in both fur and feces (S6 Fig). In conclusion, we found no evidence for differences in corticosterone levels between immune-deficient Rag^-/-^ and immune-competent Rag^+/-^ mice.

## Discussion

We used a multi-parametric analysis to evaluate animal distress and found no indication for clear differences in well-being between immune-deficient and immune-competent animals. Other studies using the same protocol to quantify distress in other settings found that non-invasive methods such as measuring body weight, corticosterone metabolites and nesting behavior are suitable and may be superior to other physiological read outs such as heart rate, body temperature and activity measured by telemetry [12, 13]. Our results seem to contrast earlier studies of Rag-deficient mice suggesting differences in behavior [7] or sense of smell [5]. But the interpretation of these results is difficult, because the experiments suggesting a difference included purchased wild type control mice instead of littermates. Thus, environmental factors (e.g. the microbiome) and minor genetic differences of separately propagated inbred lines cannot be excluded. Another study found a shortened life span for Rag-deficient mice and had used littermate controls. However, in this study mice were potentially infected with *Pasteurella pneumotropica* and *Helicobacter pylori*, which may affect life span of the animals due to inflammation [19]. Since we terminated the experiment at nine months, the typical life span in the breeding colony, we cannot exclude that even under SPF according to the Federation of European Laboratory Animal Science Associations (FELASA), life span may be affected at a later stage.

In summary, some of the tests used show very weak signals, e.g. 1–2% of Rag mice have a slight reaction in the MGS. While we cannot formally exclude that pain masking behavior has interfered with this read out, we think it is unlikely that we have overlooked exposure to a major and chronic pain inducing factor, since the majority of other tests and parameters did not show differences. Furthermore, we have observed the animals over a longer period of time and pain masking behavior over a period of 9 months in case of severe pain, we consider unlikely. When comparing weight, heterozygous Rag mice seemed slightly heavier, even though the difference was not statistically significant. The small differences we found also in histopathology or weight are in the range of those found e.g. between different immune-competent inbred strains of mice. For stress hormones there are no differences between immune-deficient and–competent mice but there was a difference for age or sex. Therefore, in our judgement, there is no indication of an increased burden or severity of the tested immune-deficient mouse lines, when kept under SPF conditions according to FELASA. While our study has shown that even the two severe immune-deficient mouse lines analyzed, under the particular local conditions in our facility do not show signs of burden, considering the complex nature of the interactions of microorganisms with the immune system, there is probably not an absolute threshold of microorganisms, commensals or pathogens for all immune-deficient mice but rather depending on the type of immune-deficiency and interactions with the local microorganisms will decide whether the well-being of a particular immune-deficient mouse strain is affected. Therefore, further studies with different mouse lines in different facilities and maybe using larger cohorts of mice are warranted to test if these findings are more generally applicable. Importantly, European regulatory authorities currently attribute severity to all immune-deficient mice (also those kept in SPF conditions). This assessment has not been substantiated by facts. It leads to considerably more administrative work, slowing down research and tying up administrative resources. From an immunological point of view, it is an oversimplification to use a term that covers a wide variety of conditions, to specifically ascribe suffering to animals carrying alterations in their immune system. It seems more expedient to only report to the regulatory authorities if obvious severity is observed, instead of using "immune-deficient" as an umbrella term falsely equating it with distress and suffering. Many types of immune-deficiency show a mild, almost undetectable phenotype (e.g. CCR5 deficiency) and even severe deficiencies such as the Rag deficiency are not associated with obvious suffering under SPF conditions. Severity has also been postulated based on the theoretical and legal consideration that SPF housing is a refinement. From a scientific point of view, housing in SPF and working under SPF conditions for both immune-competent and immune-deficient mice is clearly not a “refinement”. In order to exclude artifacts caused by pathogens, SPF conditions are a *conditio sine qua non* in most experimental settings.

Taken together, animal well-being above all is ethically imperative and it is also an important issue that immunologists take seriously because it improves standardization and reproducibility of research results. However, we propose to base decisions on animal well-being and severity on objective parameters determined by laboratory animal science.

## Supporting information

S1 Fig Study design and test schedule of the main study protocol. Schematic representation of the plan and test procedures of the main study.
(PDF)

S2 Fig Statistical evaluation of corticosterone and weight development of the main study cohort. (PDF)

S3 Fig Testosterone levels in immune-deficient and -competent mice. Fur testosterone levels measured from the same samples that corticosterone was determined of mice from the main study.
(PDF)

S4 Fig Nest building behavior of mice from the main study. (PDF)

S5 Fig Corticosterone levels in Rag^+/+^ and Rag^-/-^ and FVB/N mice. Fur corticosterone levels measured from non-littermate mice from the breeding colony and the unrelated non-C57BL/6 strain FVB/N.
(PDF)

S6 Fig Corticosterone levels in Rag^+/-^ and Rag^-/-^ on a C3H or C57BL/6 genetic background. Fur corticosterone and feces corticosterone metabolite levels measured from littermate mice.
(PDF)

S1 Table Mice included in the main study protocol. Overview of the main study protocol (numbers, sex, strain of mice and average age during fur sampling).
(PDF)

S2 Table Mouse grimace scale analysis. Summary of the facial expression symptom score (grimace scale) of mice from the main study.
(PDF)

S3 Table Summary of histopathological analysis. Overview over histopathological analysis of spleen, heart, intestine, lung, liver and kidney of the mice from the main study.
(PDF)

S4 Table Mice sampled for additional corticosterone analysis. Overview of sex, number, and strain of mice that where analyzed for corticosterone (metabolites) only at one time point, in addition to the prospective longitudinally analyzed main study cohort.
(PDF)

S5 Table Primary data from this study. Excel spreadsheets with primary data of this study.
(XLSX)
