# Supplementary figures and images for: Stress hormones or general well-being are not altered in immune-deficient mice lacking either T- and B- lymphocytes or Interferon gamma signaling if kept under specific pathogen free housing conditions
Source: PLoS One. 2020 Sep 30;15(9):e0239231. doi: 10.1371/journal.pone.0239231 (PMC7526874; doi:10.1371/journal.pone.0239231)

Study design: Test schedule of the main study cohort

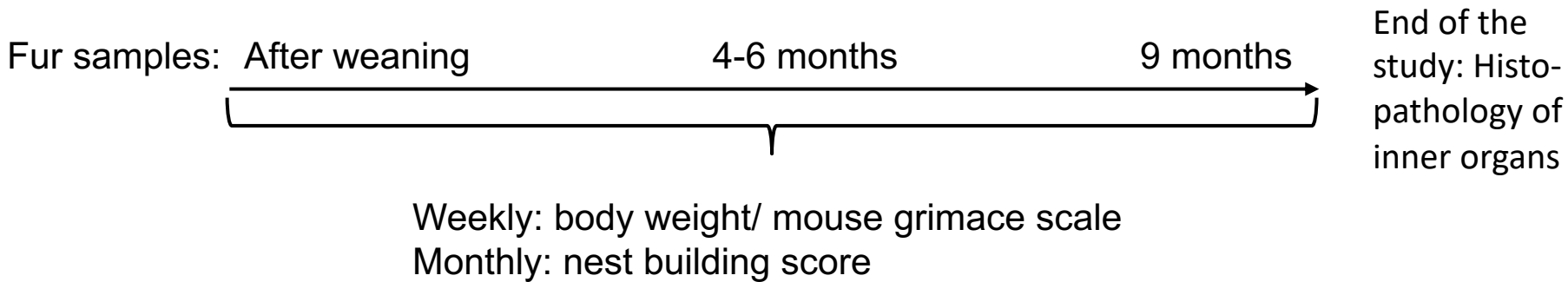

Supplement: S1 Fig — Schematic representation of the plan and test procedures of the main study. (PDF) [file pone.0239231.s001.pdf]
