# Supplementary material for: Stress hormones or general well-being are not altered in immune-deficient mice lacking either T- and B- lymphocytes or Interferon gamma signaling if kept under specific pathogen free housing conditions
Source: PLoS One. 2020 Sep 30;15(9):e0239231. doi: 10.1371/journal.pone.0239231 (PMC7526874; doi:10.1371/journal.pone.0239231)
Supplement: S2 Fig — (PDF) [file pone.0239231.s002.pdf]

Supporting Figure 2

Statistical analysis of corticosterone and weight analysis

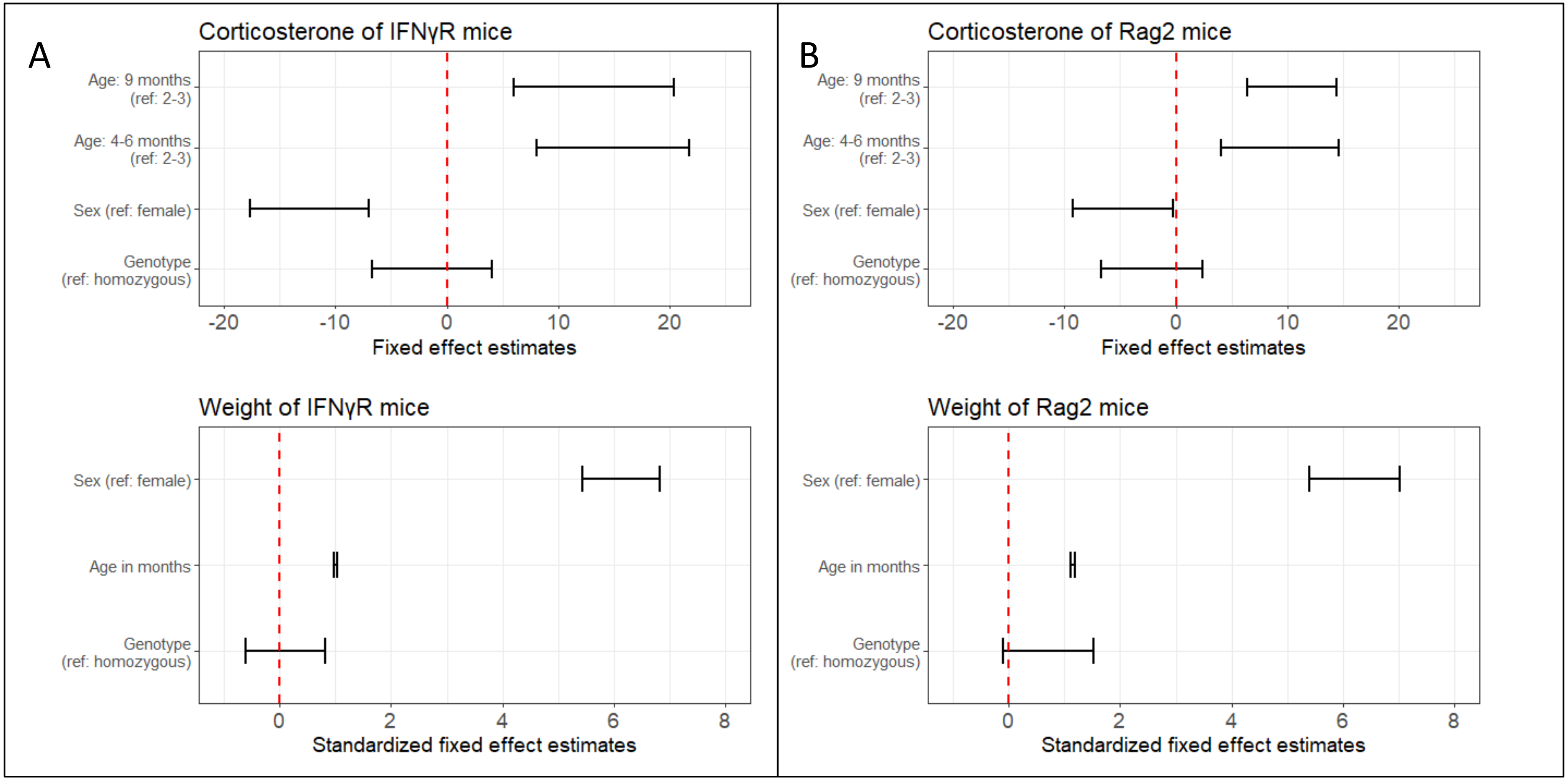

95% confidence intervals for the fixed effect estimates from linear mixed effects models. Models were fitted for IFNγR (A) and Rag (B), separately. The outcome variable of the model was corticostesterone level and weight, respectively. All independent variables used in the model are displayed. For categorical variables, the reference category is in brackets. In all models, a random intercept for each individual mouse was used to account for repeated measures. The lme()-function from the R package nlme [17] was used to fit the models. The confidence intervals are based on normal approximation and were calculated using the interval()-function from the same R package. For a more detailed interpretation including p-values see text below (next page).

To model weight and corticosterone over time, linear mixed models were fitted using the lme()-function in the R package nlme (Pinheiro et al. 2020). A random intercept for individual mice was specified. Time, genotype and sex were included as independent variables. Corticosterone was measured at three time points, therefore time was included as a factor, while for weekly weight measurements time was considered as continuous variable.

Corticosterone:

For IFNgR-mice, there were significant effects for months 4-6 (β=14.92, 95%-CI [8.05; 21.79], $p<0.001$, reference category (ref.) 2-3 months), for month 9 (β=13.19, 95%-CI=[5.97; 20.41], $p=0.002$, ref. 2-3 months) and for sex (β=-12.34, 95%-CI=[-17.69; -6.99], $p<0.001$, ref. female). The effect of the genotype was not significant (β=-1.34, 95%-CI=[-6.73;4.05], $p=0.619$, ref. homozygote).

For Rag2-mice, there were significant effects for months 4-6 (β=9.33, 95%-CI [4.08; 14.58], $p=0.001$, reference category (ref.) 2-3 months), for month 9 (β=10.42, 95%-CI=[6.42; 14.41], $p<0.001$, ref. 2-3 months) and for sex (β=-7.71, 95%-CI=[-9.21; -0.21], $p=0.041$, ref. female). The effect of the genotype was not significant (β=--2.17, 95%-CI=[-6.71;2.38], $p=0.338$, ref. homozygote).

Body weight:

For IFNgR-mice, there were significant effects of time in months (β=1.15, 95%-CI [1.12; 1.18], $p<0.001$) and for sex (β=6.20, 95%-CI=[5.39; 7.01], p=<0.001, ref. female). The effect of the genotype was not significant (β=0.70, 95%-CI=[-0.11; 1.51], $p=0.087$, ref. homozygote).

For Rag2-mice, there were significant effects of time in months (β=1.01, 95%-CI [0.98; 1.03], $p<0.001$) and for sex (β=6.13, 95%-CI=[5.43; 6.83], p=<0.001, ref. female). The effect of the genotype was not significant (β=0.10, 95%-CI=[-0.60; 0.81], $p=0.77$, ref. homozygote).
