# Supplementary material for: Stress hormones or general well-being are not altered in immune-deficient mice lacking either T- and B- lymphocytes or Interferon gamma signaling if kept under specific pathogen free housing conditions
Source: PLoS One. 2020 Sep 30;15(9):e0239231. doi: 10.1371/journal.pone.0239231 (PMC7526874; doi:10.1371/journal.pone.0239231)
Supplement: S3 Fig — Fur testosterone levels measured from the same samples that corticosterone was determined of mice from the main study. (PDF) [file pone.0239231.s003.pdf]

Supporting Figure 3

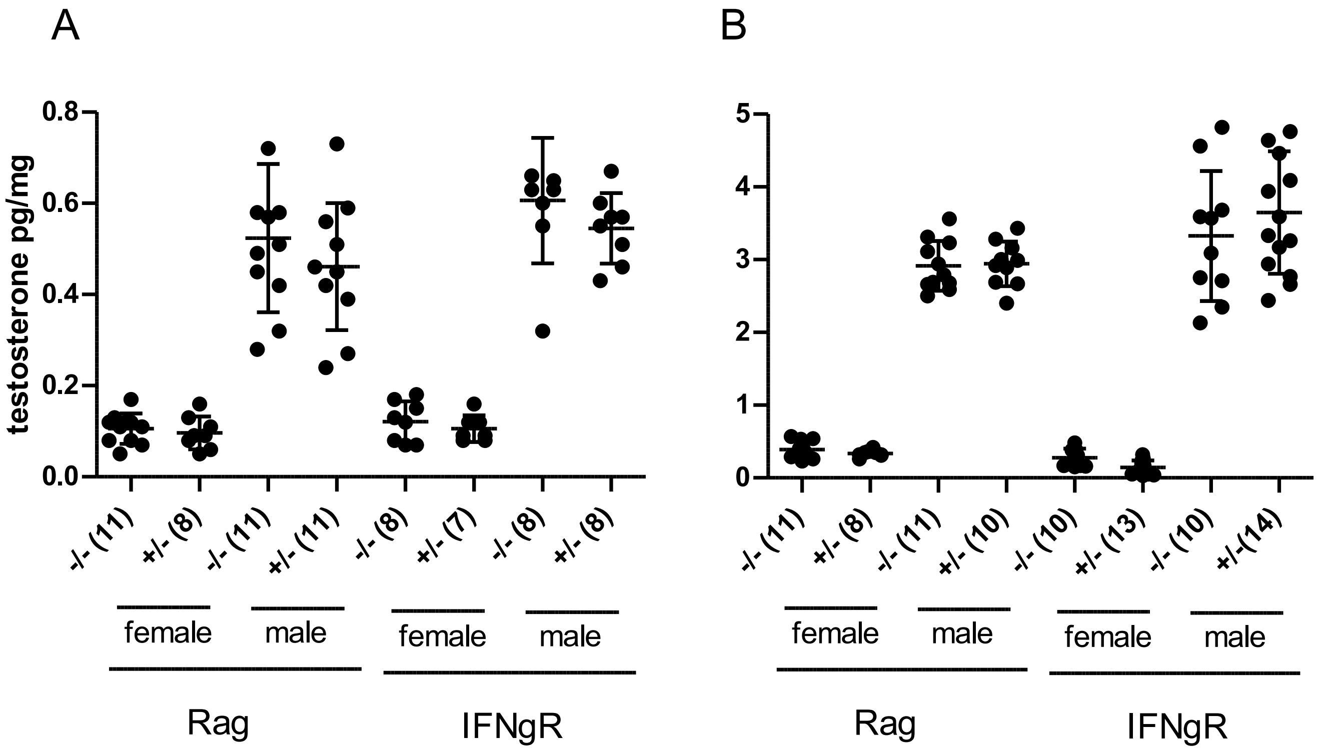

Testosterone levels in immune-deficient and -competent mice. Fur samples of male and female mice with the respective genotypes (IFNγR$^{+/-}$ IFNγR$^{-/-}$,Rag$^{+/-}$ and Rag$^{-/-}$) were harvested from **A,** young 2-3 months and **B,** 9 months old mice. Samples were analysed for testosterone by LC/MS. Each dot represents one mouse. Note that analysis was performed in parallel with corticosterone from the same samples as shown in Figure 2. In parentheses the number of samples tested (n) is given. We did not perform statistical analysis (e.g. by providing a p-value) between male and female mice because the difference between the sexes is an established fact. We rather provide this data to show that our test can sensitively detect steroid hormones in the fur samples.
