# Supplementary material for: Stress hormones or general well-being are not altered in immune-deficient mice lacking either T- and B- lymphocytes or Interferon gamma signaling if kept under specific pathogen free housing conditions
Source: PLoS One. 2020 Sep 30;15(9):e0239231. doi: 10.1371/journal.pone.0239231 (PMC7526874; doi:10.1371/journal.pone.0239231)
Supplement: S4 Fig — (PDF) [file pone.0239231.s004.pdf]

Supporting Figure 4

A

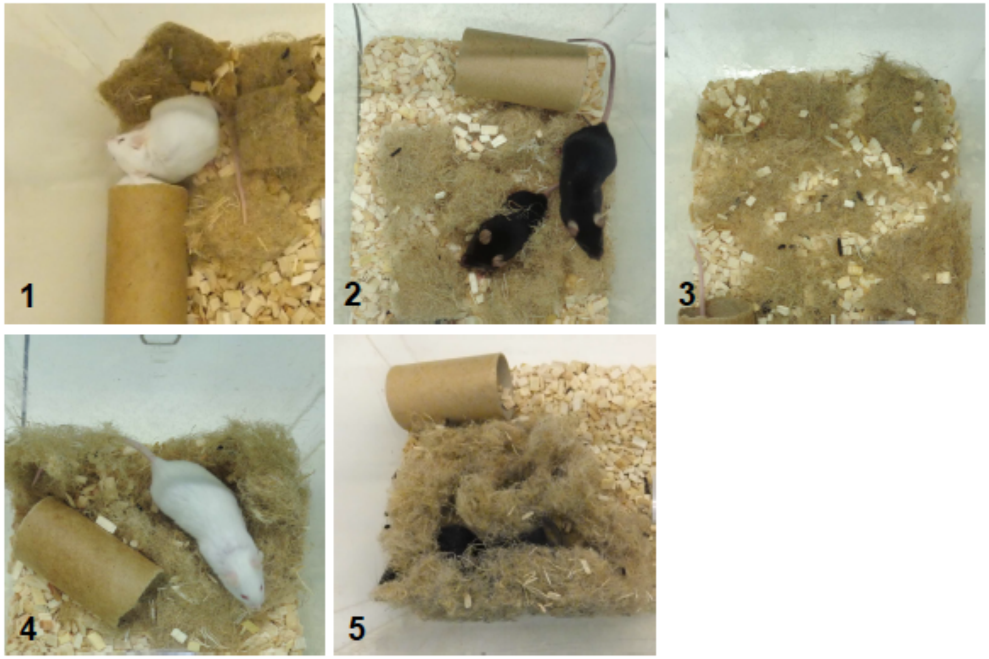

B

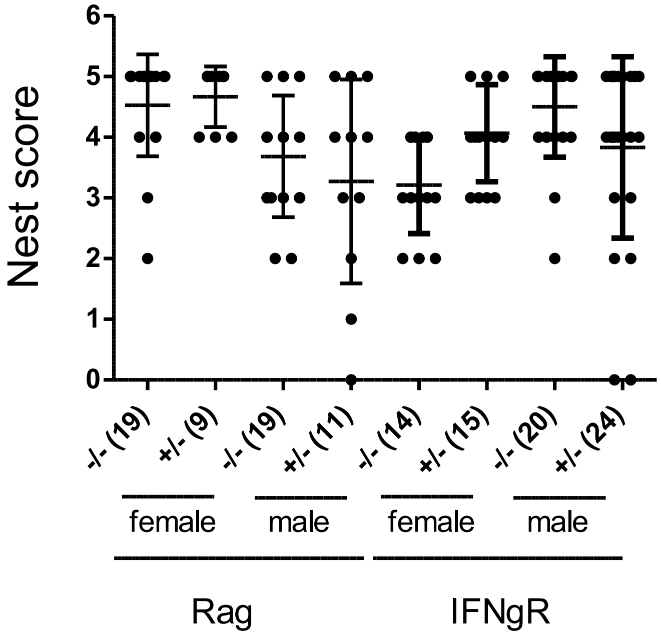

Nest building behavior. Three days after placing the nestlets into the cage, nests were scored. **A** examples of nests on the 5-point scale (1 = more than 90 % of the nestlet intact; 2 = 50–90 % intact; 3 = 50–90 % shredded nestlet; 4 = more than 90 % shredded but flat nest, i.e. less than 50 % of its circumference being higher than the body height of a curled-up mouse; 5 = more than 90 % shredded and high nest, i.e. more than 50 % of its circumference being higher than the body height of a curled-up mouse). **B** summary of monthly gathered nests scores. Each dot represents one nest scored at one point in time. Number of nests/cages scored in each group is indicated in parentheses below the x-axis.
