# Supplementary material for: Stress hormones or general well-being are not altered in immune-deficient mice lacking either T- and B- lymphocytes or Interferon gamma signaling if kept under specific pathogen free housing conditions
Source: PLoS One. 2020 Sep 30;15(9):e0239231. doi: 10.1371/journal.pone.0239231 (PMC7526874; doi:10.1371/journal.pone.0239231)
Supplement: S5 Fig — Fur corticosterone levels measured from non-littermate mice from the breeding colony and the unrelated non-C57BL/6 strain FVB/N. (PDF) [file pone.0239231.s005.pdf]

Supporting Figure 5

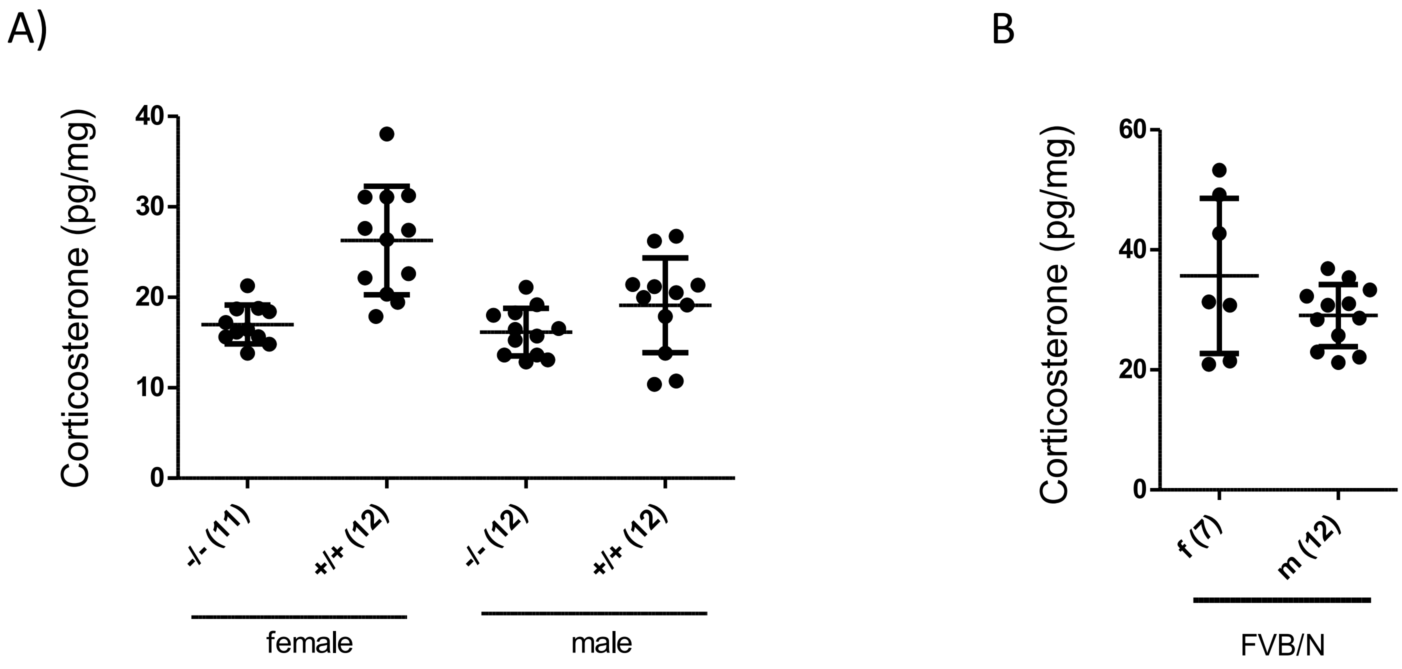

Corticosterone levels in non-littermate A) Rag$^{+/+}$ and Rag$^{-/-}$ mice on a C57BL/6J background and B) as a control wildtype mice on an FVB/N background. Fur samples of male and female A) Rag$^{+/+}$ and Rag$^{-/-}$ (none-littermate) or B) FVB/N mice from the breeding colony, were harvested at 9 months of age and were analysed for corticosterone by LC/MS. Each dot represents one mouse. B) f, female and m, male. In parentheses the number of samples tested (n) is given.
