# Supplementary material for: Stress hormones or general well-being are not altered in immune-deficient mice lacking either T- and B- lymphocytes or Interferon gamma signaling if kept under specific pathogen free housing conditions
Source: PLoS One. 2020 Sep 30;15(9):e0239231. doi: 10.1371/journal.pone.0239231 (PMC7526874; doi:10.1371/journal.pone.0239231)
Supplement: S6 Fig — Fur corticosterone and feces corticosterone metabolite levels measured from littermate mice. (PDF) [file pone.0239231.s006.pdf]

Supporting Figure 6

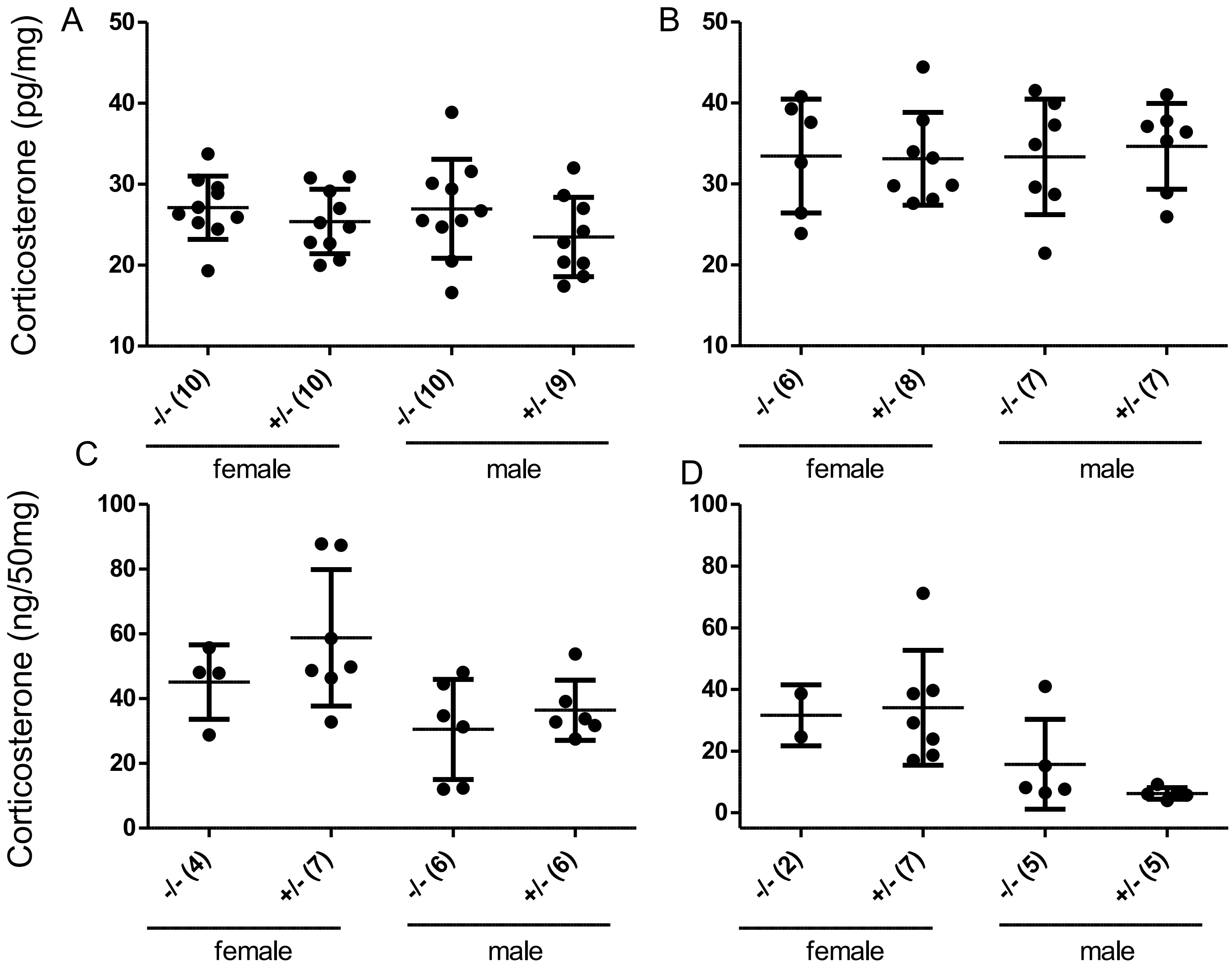

Corticosterone levels in C3H and C57l/6 Rag$^{+/-}$ and Rag$^{-/-}$ mice Fur (**A, B**) and feces (**C, D**) samples of male and female Rag$^{+/-}$ and Rag$^{-/-}$ littermate mice on a C3H (**A,C**) or C57BL6 (**B, D**) genetic background, were harvested at 4 to 6 months of age and were analysed for corticosterone metabolites (fur by LC/MS and feces by EIA). Each dot represents one mouse. Please note that corticosterone samples and values for fur of B6 mice (panel **B**) are identical as those shown in Figure 2 and are just included for better comparison. In parentheses the number of samples tested (n) is given. Sample sizes especially for faeces analysis are rather small and therefore strict statistical comparisons may not be meaningful. However, this data shows a very similar pattern as the Figure 2, and therefore supports our main findings.
