# Supplementary material for: Stress hormones or general well-being are not altered in immune-deficient mice lacking either T- and B- lymphocytes or Interferon gamma signaling if kept under specific pathogen free housing conditions
Source: PLoS One. 2020 Sep 30;15(9):e0239231. doi: 10.1371/journal.pone.0239231 (PMC7526874; doi:10.1371/journal.pone.0239231)
Supplement: S1 Table — Overview of the main study protocol (numbers, sex, strain of mice and average age during fur sampling). (PDF) [file pone.0239231.s007.pdf]

Strain	Males ♂	Age at Fur sampling (in weeks ± SD)	Females ♀	Age at fur sampling (in weeks +/- SD)
**B6. Rag$^{-/-}$**	12	S1: 7.8 ± 0. 4<br>S2： 21. 8 ± 3. 8<br>S3: 38.7 ± 1. 2	11	S1: 7.6 ± 0. 6<br>S2： 20. 8 ± 3. 7<br>S3: 38.3 ± 1. 2
**B6.Rag$^{+/-}$**	11	S1: 7.2 ± 0. 8<br>S2： 20. 4 ± 4. 1<br>S3: 37.8 ± 1. 1	8	S1: 6.6 ± 0. 5<br>S2： 17. 6 ± 0. 5<br>S3: 37.6 ± 0. 5
**B6.IFNγR$^{-/-}$**	10	S1: 8.5 ± 2. 3<br>S2： 23. 4 ± 2. 8<br>S3: 39.2 ± 1. 6	10	S1: 8.6 ± 2. 2<br>S2： 23. 8 ± 3. 0<br>S3: 39.2 ± 1. 6
**B6.IFNγR$^{+/-}$**	14	S1: 9.3 ± 2. 7<br>S2： 21. 6 ± 3. 1<br>S3: 38.6 ± 1. 7	14	S1: 9.4 ± 2. 9<br>S2： 20. 5 ± 1. 6<br>S3: 38.4 ± 1. 7

Supporting Table 1: Mice included in the main study protocol and age at fur sampling

S1: Sample1 after weaning; S2: Sample 2 at ca. 6 months; S3: Sample 3 at the end of the experiment ca. 9 months
