# Supplementary material for: Stress hormones or general well-being are not altered in immune-deficient mice lacking either T- and B- lymphocytes or Interferon gamma signaling if kept under specific pathogen free housing conditions
Source: PLoS One. 2020 Sep 30;15(9):e0239231. doi: 10.1371/journal.pone.0239231 (PMC7526874; doi:10.1371/journal.pone.0239231)
Supplement: S2 Table — Summary of the facial expression symptom score (grimace scale) of mice from the main study. (PDF) [file pone.0239231.s008.pdf]

Supporting Table 2: Mouse grimace scale analysis

MGS symptom score	$Rag^{-/-}$	$Rag^{+/-}$	$IFN\gamma R^{-/-}$	$IFN\gamma R^{+/-}$
not present (score 0)	13716	11000	12990	18187
moderately present (score 1)	203	119	29	30
obviously present (score 2)	1	0	0	3

Facial expression (grimace sacle) was judged weekly over a 9 months period by four persons blinded to the experimental setup. Mice were scored for orbital tightening, nose bulge, cheek bulge, ear position, and whisker change on a scale from 0 to 2 (0 = not present, 1 = moderately present, 2 = obviously present).
