# Supplementary material for: Stress hormones or general well-being are not altered in immune-deficient mice lacking either T- and B- lymphocytes or Interferon gamma signaling if kept under specific pathogen free housing conditions
Source: PLoS One. 2020 Sep 30;15(9):e0239231. doi: 10.1371/journal.pone.0239231 (PMC7526874; doi:10.1371/journal.pone.0239231)
Supplement: S3 Table — Overview over histopathological analysis of spleen, heart, intestine, lung, liver and kidney of the mice from the main study. (PDF) [file pone.0239231.s009.pdf]

Supporting Table 3: Summary of histopathological analysis

	Rag2 $^{+/-}$	Rag2 -/-	IFNγR $^{+/-}$	IFNγR $^{-/-}$	FVB
Lymphoma	(1/17)	(1/22)	(0/22)	(0/19)	(0/16)
CPN	(11/17)	(17/22)	(8/22)	(4/19)	(0/16)
AMP	(0/17)	(6/22)	(0/22)	(0/19)	(0/16)
PT2H	(2/17)	(2/22)	(1/22)	(4/19)	(0/16)
BALT	(1/17)	(0/22)	(1/22)	(1/19)	(0/16)
IP	(1/17)	(1/22)	(0/22)	(0/19)	(0/16)
Hepatitis	(0/17)	(0/22)	(0/22)	(0/19)	(1/16)

Shown in parentheses (number of mice with lesion / number of mice analyzed). **CPN** (chronic progressive nephropathy) is the single most important renal disease in mice. CPN has been specifically described in the C57BL/6 genetic background but also occurs in other strains. The pathogenesis and etiology of CPN is unknown, but factors such as strain, sex, age, diet, and hormones may modulate its occurrence and severity. **BALT** (Broncus Assoaciated Lymphoid Tissue) -hyperplasia, **PT2H** Pneumocyte Typ II-Hyperplasia, **AMP** Acidophilic Macrophage Pneumonia are lesions of the lung. BALT and PT2H both are caused by subacute to chronic irritation or damage of the alveolar epithelium. AMP is a sporadic, idiopathic pulmonary disease of laboratory mice, commonly affecting C57BL/6 mice. **IP** Interstitial pneumonia.
