# Supplementary material for: Stress hormones or general well-being are not altered in immune-deficient mice lacking either T- and B- lymphocytes or Interferon gamma signaling if kept under specific pathogen free housing conditions
Source: PLoS One. 2020 Sep 30;15(9):e0239231. doi: 10.1371/journal.pone.0239231 (PMC7526874; doi:10.1371/journal.pone.0239231)
Supplement: S4 Table — Overview of sex, number, and strain of mice that where analyzed for corticosterone (metabolites) only at one time point, in addition to the prospective longitudinally analyzed main study cohort. (PDF) [file pone.0239231.s010.pdf]

Supporting Table 4: Mice sampled for additional corticosterone analysis

Strain	Males ♂ (fur samples/feces samples)	Females ♀ (fur samples/feces samples)
FVB/N	(12/-)	(7/-)
C57BL/6	(12/-)	(12/-)
C57BL/6 $Rag^{-/-}$	(12/-)	(11/-)
C3H. $Rag^{-/-}$	(10/6)	(10/4)
C3H. $Rag^{+/-}$	(9/6)	(10/7)

- No samples taken
